# Aspirin and non-steroidal anti-inflammatory drugs use reduce gastric cancer risk: A dose-response meta-analysis

**DOI:** 10.18632/oncotarget.13591

**Published:** 2016-11-25

**Authors:** Xuan-zhang Huang, You Chen, Jian Wu, Xi Zhang, Cong-cong Wu, Chao-ying Zhang, Shuang-shuang Sun, Wen-jun Chen

**Affiliations:** ^1^ Department of Chemotherapy and Radiotherapy, The Second Affiliated Hospital and Yuying Children's Hospital of Wenzhou Medical University, Wenzhou City 325027, P.R. China; ^2^ The Wenzhou Dental Hospital, Wenzhou City 325027, P.R. China

**Keywords:** aspirin, non-steroidal anti-inflammatory drugs, gastric cancer, chemoprevention, meta-analysis

## Abstract

**Background:**

The association between non-steroidal anti-inflammatory drugs (NSAIDs) and gastric cancer (GC) risk is controversial. The aim of this study is to evaluate the chemopreventive effect of NSAIDs for GC.

**Methods:**

A literature search was performed for relevant studies using the PubMed and Embase database (up to March 2016). Risk ratios (RRs) and 95% confidence intervals (CIs) were used as the effect measures. The dose–response analysis and subgroup analysis were also performed.

**Results:**

Twenty-four studies were included. Our results indicated that NSAIDs could reduce GC risk (any NSAIDs: RR=0.78, 96%CI=0.72-0.85; aspirin: RR=0.70, 95%CI=0.62-0.80; non-aspirin NSAIDs: RR=0.86, 95%CI=0.80-0.94), especially for non-cardia GC risk. Moreover, the dose-response analysis indicated the risk of GC decreased by 11% and 5% for 2 years increment of any NSAIDs and aspirin use, respectively. There were nonlinear relationships between the frequency of any NSAIDs use and aspirin use and GC risk (P for non-linearity<0.01), with a threshold effect of 5 times/week. A monotonically decreasing trend was observed only for the frequency of less than 5 times/week.

**Conclusions:**

Our results indicate that NSAIDs is inversely associated with GC risk, especially for non-cardia GC risk. NSAIDs use may become a feasible approach to prevent GC.

## INTRODUCTION

Gastric cancer (GC) is one of the most frequently diagnosed cancer worldwide [1]. Although a decline in GC incidence has been observed due to the primary prevention strategies including increased consumption of fresh fruits and vegetables, decreased intake of salt-preserved foods, and reduction in *Helicobacter pylori* (*H. pylori*) infection and smoking, GC has a unsatisfying prognosis and still remains the third leading cause of cancer deaths [1, 2]. Thus, more effective prevention strategies for GC are still urgently needed.

Evidences have revealed that gastric carcinogenesis develops from a complex multi-factorial and multi-stage process [3]. And studies report that inflammation is a participant in the neoplastic process [4]. The expression of cyclooxygenase (COX)-2 is increase in GC, suggesting that COX-2 may promote early gastric carcinogenesis [5, 6]. Non-steroidal anti-inflammatory drugs (NSAIDs), including nonselective NSAIDs (i.e., aspirin) and selective NSAIDs (i.e., celecoxib) can inhibit inflammation status through suppressing COX-2. Therefore, NSAIDs as a potential cost-effective chemoprevention pathway against GC gains the most recent interest. However, several epidemiological and clinical studies evaluating the association between NSAIDs and GC risk obtain inconsistent results [7–10]. Several meta-analyses have been performed to assess the association [11–13]. However, no meta-analyses conduct a dose–response analysis to evaluate the relationship between duration and frequency of NSAIDs use and GC risk, and furthermore these published meta-analyses could not conclude that whether the chemopreventive effect is different according to different tumor sites (cardia and non-cardia GC) because cardia and non-cardia GC have different risk factors, tumor characteristics, and biological behavior [14–16]. Besides, there is a lack of reasonable and in-depth subgroup analyses based on different types of NSAIDs use. Indeed, meta-analyses by Tian et al., Abnet et al. and Bosetti et al do not evaluate the duration and frequency of NSAIDs use [11–13], and meta-analysis by Bosetti et al. only assessed aspirin use and do not analyzed the effects of different tumor site [13].

Thus, the aims of this study are to assess the dose–response association between NSAIDs and GC risk and whether the chemopreventive effect of NSAIDs differed according to tumor site (cardia and non-cardia GC), medication type (aspirin and non-aspirin NSAIDs), and duration and frequency of NSAIDs use.

## RESULTS

### Study selection and study characteristics

3482 studies were initially identified in the literature search. 3319 studies were excluded after reviewing titles and abstracts. After full-text review of the remaining 164 studies, 140 studies were excluded due to irrelevant data of interest and duplicated studies. Finally, 24 full-text studies were included (Supplementary Figure S1) [7–10, 12, 17–35].

The 24 studies were published between 1993 and 2014, and were conducted in the USA, Korea, China, the UK, Italy, Sweden, Denmark, and Russia. Of the eligible studies, fourteen studies assess the chemoprevention of NSAIDs that was not limited to aspirin [9, 12, 22–27, 29–34], and ten studies assess the chemoprevention of aspirin only [7, 8, 10, 17–21, 28, 35]. Moreover, the site of GC was not otherwise specified (GC NOS) in sixteen studies [7–10, 17–21, 27–30, 32, 33, 35], seven studies provided results for cardia and non-cardia GC separately [12, 22–26, 31], one studies provided results for GC NOS and non-cardia GC [34]. The detailed characteristics are showed in Table 1, Supplementary Table S2, and Supplementary Table S3.

**Table 1:** Baseline characteristics and design variables of the included studies

Article	Year	Country	Study type	Sample	Drug type	Definition of use	GC type	Adjusted variables	Study quality	Findings
Kim	2015	Korea	PBS cohort	11715	Aspirin	100mg for ≥6 months	GC NOS	NR	7	Longer duration of aspirin use reduced GC NOS risk
Wang	2015	China	HBS CCS	525	Aspirin	≥1/week for ≥1 year	GC NOS	Gender, marriage, education, resident district, history of diabetes, BMI, cigarette smoking, alcohol drinking, and helicobacter pylori	7	Aspirin use reduced GC NOS risk
Gong	2014	Korea	HBS CCS	654	Aspirin	NR	GC NOS	NR	6	Aspirin use reduced GC NOS risk
Cook	2013	USA	PBS random trial	39876	Aspirin	100 mg of alternate-day	GC NOS	NR	7	Aspirin use could not reduce GC NOS risk
Jacobs	2012	USA	PBS cohort	100139	Aspirin	Daily	GC NOS	Age, sex, race, education, smoking, BMI, physical activity, history of heart disease, stroke, diabetes, hypertension, cholesterol-lowering drug use, aspirin use, NSAIDs use, and history of colorectal endoscopy	8	Aspirin use reduced GC NOS risk
Lee	2012	Korea	HBS CCS	1966	Aspirin	NR	GC NOS	NR	6	Aspirin use reduced GC NOS risk
Rothwell	2011	UK	PBS random trials	10502	Aspirin	Daily	GC NOS	NR	6	Longer duration of aspirin use reduced GC NOS risk
Bertuccio	2010	Italy	HBS CCS	772	Aspirin	≥1/week for ≥6 months	GC NOS	Period of interview, education, BMI, tobacco smoking, and family history of gastric cancer	7	Aspirin use could not reduce GC NOS risk
Wu	2010	Taiwan	PBS cohort	52161	Any NSAIDs	≥28/month for ≥6 months	GC NOS	NR	7	Aspirin use reduced GC NOS risk
Figueroa	2009	USA	PBS CCS	1062	Any NSAIDs, aspirin	≥1/week for ≥6 months	Cardia GC	Center, age, race, gender, cigarette smoking, GERD, proxy interview, and BMI	7	Aspirin, non-aspirin, and NSAIDs use reduced non-cardia GC risk but not cardia GC risk
Epplein	2009	USA	PBS cohort	169292	Any NSAIDs, aspirin, non-aspirin	≥2/week for ≥1 months	Cardia, non-cardia	Age, sex, ethnicity, smoking, BMI, and alcohol consumption	7	Aspirin and NSAIDs use reduced non-cardia GC risk but not cardia GC risk
Abnet	2009	USA	PBS cohort	311115	Any NSAIDs, aspirin, non-aspirin	≥1/month for ≥1 year	Cardia, non-cardia	Age, sex, cigarette smoking status, alcohol, education, fruit intake, vegetable intake, BMI, total energy intake, and physical activity	7	Aspirin, non-aspirin, and NSAIDs use reduced non-cardia GC risk but not cardia GC risk
Duan	2008	USA	PBS CCS	2074	Any NSAIDs, aspirin, non-aspirin	≥2/week for ≥1 month	Cardia, non-cardia	Age, sex, race, birthplace, education, smoking status, BMI, UGI history, and antacid use	7	Longer duration of aspirin and NSAIDs use reduced non-cardia GC risk but not cardia GC risk
Fortuny	2007	USA	PBS CCS	2972	Any NSAIDs, aspirin, non-aspirin	≥1 prescriptions	Cardia, non-cardia	Age, sex, HMO, years of enrollment in the HMO, race, and use of drug classes other than the studied one	7	Aspirin and NSAIDs use reduced non-cardia GC risk but not cardia GC risk, non-aspirin NSAIDs could not reduce both cardia and non-cardia GC risk
Trivers	2005	USA	PBS CCS	615	Any NSAIDs	≥1/week for 6 months	Cardia, non-cardia	NR	6	NSAIDs use could not reduce both cardia and non-cardia GC risk
Lindblad	2005	Sweden	PBS CCS	11023	Aspirin, non-aspirin	any use	GC NOS	Sex, age, smoking, alcohol consumption, BMI, calendar year, and UGI disorders	7	Longer duration of non-aspirin NSAIDs use reduced GC NOS risk, aspirin use could not reduce GC NOS risk
Ratnasinghe	2004	USA	PBS cohort	22834	Aspirin	any use	GC NOS	BMI, sex, race, poverty index, education and smoking	6	Aspirin use could not reduce GC NOS risk
Sorensen	2003	Denmark	PBS cohort	172057	Any NSAIDs	≥1 prescriptions	GC NOS	NR	6	NSAIDs use could not reduce GC NOS risk
Nomura	2003	USA	HBS CCS	746	NSAIDs	≥2/week for ≥3month	GC NOS	Sex, age, and ethnicity	7	NSAIDs use reduced GC NOS risk
Akre	2001	Sweden	PBS CCS	1732	Aspirin, non-aspirin	≥1/month	GC NOS, cardia, non-cardia	Age, gender and socioeconomic status	7	Aspirin use could not reduce both cardia and non-cardia GC risk
Langman	2000	UK	PBS CCS	2450	Any NSAIDs	≥1 prescriptions	GC NOS	Age and smoking status	7	More NSAIDs prescriptions use reduced GC NOS risk
Coogan	2000	USA	HBS CCS	6083	NSAIDs, aspirin	≥4/week for ≥ 3 months	GC NOS	Age, sex, interview year, center, race, religion, cigarettes, family history of digestive cancer, education, and alcohol consumption	8	Aspirin use reduced GC NOS risk
Zaridze	1999	Russia	HBS CCS	1058	Any NSAIDs, aspirin	≥2/week for 6 months	GC NOS, non-cardia	Age and education	6	Aspirin and NSAIDs use reduced non-cardia GC risk but not cardia GC risk
Thun	1993	USA	PBS cohort	635031	Aspirin	≥1/month	GC NOS	Age, race, sex, BMI, number of cigarette smoked, drinks of alcohol, total dietary fat, and fruit/vegetable/grain consumption, family history of the specific cancer, any tobacco use, and ever pipe or cigar	8	Aspirin use reduced GC NOS risk

Abbreviations, BMI: Body mass index; CCS: Case-control study; GC: Gastric cancer; GERD: Gastroesophageal reflux disease; HBS: Hospital-based study; HMO: Health maintenance organization; NOS: Not otherwise specified; NR: Not reported; NSAIDs: Non-steroidal anti-inflammatory drugs; PBS: Population-based study; UGI: Upper gastrointestinal; UK: the United Kingdom; USA: the United States of America.

### NSAIDs use and GC NOS

**Figure 1:**
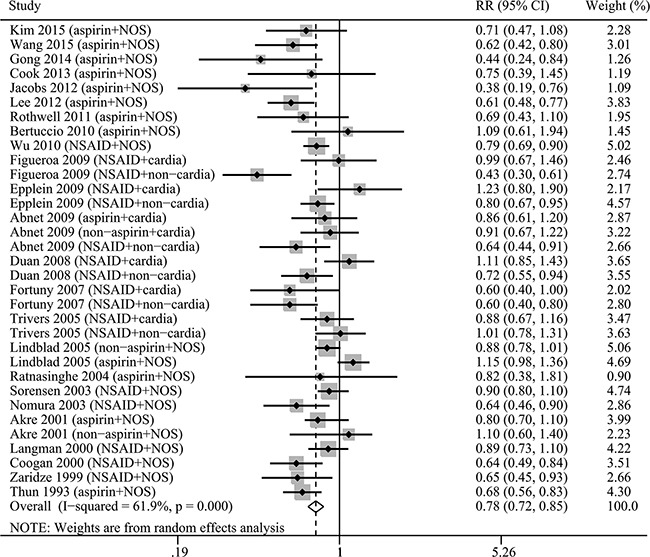
The relative risk (RR) was summarized for the relationship between any NSAIDs use and gastric cancer risk

Our results indicated that any NSAIDs use was inversely associated with GC risk (RR=0.78, 96%CI=0.72-0.85, Figure 1). Both aspirin use and non-aspirin NSAIDs use had a chemopreventive effect for GC (aspirin: RR=0.70, 95%CI=0.62-0.80; non-aspirin NSAIDs: RR= 0.86, 95%CI=0.80-0.94) (Supplementary Figure S2). As was shown by subgroup analysis on the study design, sample size, and country, the chemopreventive effect of NSAIDs on GC was confirmed in any NSAIDs group, aspirin subgroup, and non-aspirin NSAIDs subgroup (Table 2).

**Table 2:** The results of subgroup analyses for the relationship between NSAIDs and gastric cancer risk

	GC NOS		Non-cardia GC		Cardia GC	
	RR	*I^2^* (%)	RR	*I^2^* (%)	RR	*I^2^* (%)
**Any NSAIDs**						
**Total**	0.78[0.72-0.85]	61.9%	0.70[0.59-0.84]	69.6%	0.93[0.82-1.05]	16.2%
**Study type**						
Cohort	0.80[0.74-0.86]	24.5%	0.77[0.66-0.90]	15.0%	0.95[0.78-1.16]	0.0%
Case-control	0.78[0.69-0.88]	71.9%	0.68[0.53-0.88]	76.9%	0.91[0.79-1.07]	38.3%
**Study quality**						
≥7	0.78[0.70-0.86]	66.1%	0.65[0.51-0.83]	69.5%	0.91[0.79-1.05]	23.4%
<7	0.80[0.70-0.92]	52.9%	0.78[0.58-1.05]	71.6%	0.97[0.77-1.22]	39.1%
**PBS/HBS**						
PBS	0.82[0.76-0.90]	60.0%	0.73[0.60-0.87]	69.9%	0.93[0.82-1.05]	16.2%
HBS	0.64[0.56-0.72]	0.0%	/	/	/	/
**Duration of use**						
≥5 years	0.65[0.56-0.74]	49.3%	0.56[0.46-0.68]	35.9%	0.87[0.67-1.12]	40.3%
<5 years	0.78[0.67-0.90]	54.0%	0.68[0.51-0.90]	67.6%	0.84[0.66-1.08]	11.2%
**Frequency of use**						
≥7/week	0.77[0.70-0.85]	42.6%	0.64[0.52-0.80]	0.0%	1.02[0.82-1.27]	0.0%
<7/week	0.74[0.67-0.83]	0.0%	0.66[0.53-0.81]	0.0%	0.86[0.69-1.05]	0.0%
**Sample size**						
<2000	0.74[0.65-0.85]	64.7%	0.68[0.53-0.88]	76.9%	0.91[0.79-1.07]	38.3%
≥2000	0.82[0.75-0.90]	56.3%	0.77[0.66-0.90]	15.0%	0.95[0.78-1.16]	0.0%
**Country**						
Europe & America	0.81[0.74-0.88]	61.5%	0.70[0.59-0.84]	69.6%	0.93[0.82-1.05]	16.2%
Asia	0.72[0.65-0.80]	41.2%	/	/	/	/
**H. pylori status**						
H. pylori+	0.52[0.42-0.65]	0.0%	/	/	/	/
H. pylori-	0.81[0.71-0.91]	0.0%	/	/	/	/
**Aspirin**						
**Total**	0.70[0.62-0.80]	71.7%	0.64[0.53-0.78]	68.8%	0.82[0.61-1.11]	71.4%
**Study type**						
Cohort	0.72[0.65-0.80]	0.0%	0.70[0.60-0.83]	0.0%	0.91[0.70-1.19]	0.0%
Case-control	0.68[0.56-0.84]	82.1%	0.61[0.45-0.83]	78.3%	0.76[0.47-1.23]	82.2%
**Study quality**						
≥7	0.70[0.59-0.84]	77.7%	0.64[0.51-0.80]	70.7%	0.79[0.55-1.12]	76.3%
<7	0.69[0.60-0.79]	12.8%	0.63[0.43-0.92]	60.3%	/	/
**PBS/HBS**						
PBS	0.73[0.63-0.85]	74.7%	0.66[0.54-0.81]	70.7%	0.82[0.61-1.11]	71.4%
HBS	0.61[0.52-0.71]	20.1%	/	/	/	/
**Duration of use**						
≥5 years	0.73[0.63-0.84]	21.6%	0.61[0.49-0.76]	0.0%	1.01[0.78-1.31]	0.0%
<5 years	0.92[0.83-1.03]	43.0%	0.78[0.65-0.93]	17.0%	1.06[0.82-1.37]	0.0%
**Frequency of use**						
≥7/week	0.71[0.56-0.89]	53.5%	0.65[0.51-0.83]	0.0%	1.06[0.82-1.37]	0.0%
<7/week	0.73[0.64-0.82]	0.0%	0.67[0.50-0.90]	0.0%	0.76[0.56-1.05]	0.0%
**Sample size**						
<2000	0.67[0.55-0.80]	72.3%	0.61[0.45-0.83]	78.3%	0.76[0.47-1.23]	82.2%
≥2000	0.75[0.64-0.89]	67.6%	0.70[0.60-0.80]	0.0%	0.91[0.70-1.19]	0.0%
**Country**						
Europe & America	0.72[0.62-0.84]	74.1%	0.64[0.53-0.78]	68.8%	0.82[0.61-1.11]	71.4%
Asia	0.61[0.52-0.73]	0.0%	/	/	/	/
**H. pylori status**						
H. pylori+	0.53[0.36-0.77]	1.9%	/	/	/	/
H. pylori-	0.81[0.52-1.26]	0.0%	/	/	/	/
**Non-aspirin**						
**Total**	0.86[0.80-0.94]	31.0%	0.74[0.60-0.93]	58.7%	0.92[0.78-1.09]	0.0%
**Study type**						
Cohort	0.91[0.73-1.13]	51.2%	0.84[0.57-1.22]	76.8%	0.99[0.77-1.28]	16.1%
Case-control	0.84[0.77-0.93]	22.4%	0.68[0.55-0.85]	8.0%	0.87[0.70-1.10]	0.0%
**Study quality**						
≥7	0.83[0.76-0.91]	19.9%	0.68[0.57-0.81]	0.0%	0.89[0.74-1.06]	0.0%
<7	1.04[0.85-1.26]	0.0%	/	/	/	/
**Duration of use**						
≥5 years	0.84[0.62-1.12]	0.0%	0.75[0.51-1.10]	35.9%	0.97[0.62-1.51]	0.0%
<5 years	0.85[0.75-0.96]	33.0%	0.81[0.66-0.98]	14.5%	0.89[0.53-1.48]	62.3%
**Frequency of use**						
≥7/week	0.72[0.57-0.90]	10.2%	0.63[0.45-0.87]	50.0%	1.06[0.82-1.37]	0.0%
<7/week	0.79[0.64-0.97]	44.8%	0.64[0.47-0.87]	16.5%	0.76[0.56-1.05]	0.0%
**PBS/HBS**						
PBS	0.86[0.80-0.94]	31.0%	0.74[060–0.93]	58.7%	0.93[0.78-1.09]	0.0%
HBS	/	/	/	/	/	/
**Sample size**						
<2000	0.80[0.69-0.93]	26.0%	0.68[0.55-0.85]	8.0%	0.87[0.70-1.10]	0.0%
≥2000	0.89[0.81-0.98]	36.3%	0.84[0.58-1.22]	76.8%	0.99[0.77-1.28]	16.1%
**Country**						
Europe & America	0.86[0.80-0.94]	31.0%	0.74[0.60-0.93]	58.7%	0.93[0.78-1.09]	0.0%
Asia	/	/	/		/	/

Abbreviations, GC: Gastric cancer; HBS: Hospital-based study; NOS: Not otherwise specified; PBS: Population-based study; RR: Risk ratio; “/” symbol: No results due to insufficient studies

*I^2^* shows the degree of heterogeneity among studies.

**Figure 2:**
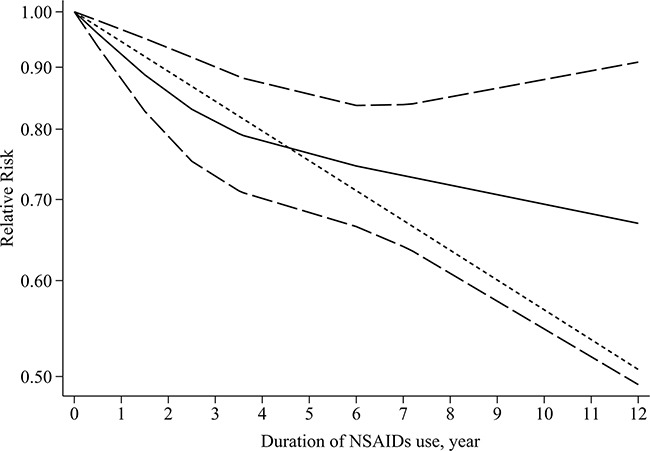
Dose–response relationship between duration of any NSAIDs use and gastric cancer risk Relative risk (RR; –––) and the corresponding 95% confidence intervals (CI; – – –) were summarized for the dose–response relationship between duration of any NSAIDs use (year) and gastric cancer risk. Data were modeled with restricted cubic spline models, where - - - - represents the linear trend.

We observed a lower GC risk in studies with longer duration of NSAIDs use compared with studies with shorter duration (RR=0.65 for ≥ 5 years VS RR=0.78 for < 5 years, Table 2 ). The results of dose–response analysis indicated that there were insignificant non-linear relationships between duration of any NSAIDs use and aspirin use and GC risk (any NSAIDs: P for non-linearity=0.12, Figure 2; aspirin: P for non-linearity= 0.27), so linear regression models were fitted (any NSAIDs: P for linear trend<0.01; aspirin: P for linear trend=0.01). And every 2 years increment for any NSAIDs use was inversely associated with GC risk (RR=0.89, 95 % CI=0.83-0.96), and aspirin use tended toward a potentially chemopreventive effect (RR=0.95, 95%CI=0.88-1.02), suggesting that similar trends were observed with linear model and subgroup analysis based on duration of NSAIDs use. A non-linear relationship existed in non-aspirin NSAIDs (P for non-linearity=0.02) and there was a maximal chemopreventive effect at duration of 5 years, with a monotonically decreasing trend for duration of less than 5 years and a increasing trend for duration of more than 5 years. Besides, a separate analysis with a linear model showed a decreased trend of GC risk for 2 years increment (RR=0.96, 95%CI=0.93-0.99).

**Figure 3:**
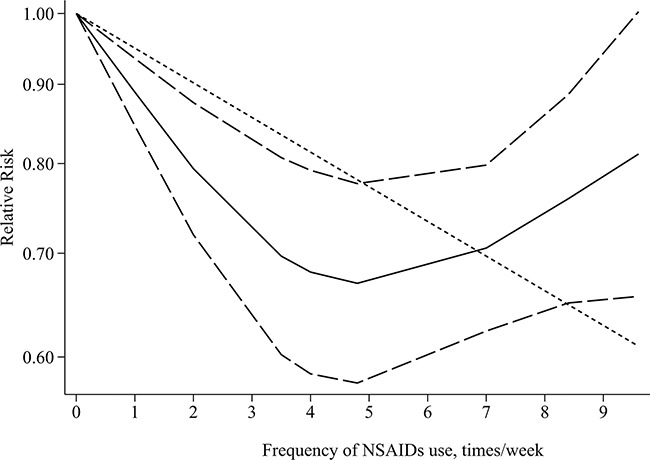
Dose–response relationship between frequency of any NSAIDs use and gastric cancer risk Relative risk (RR; –––) and the corresponding 95% confidence intervals (CI; – – –) were summarized for the dose–response relationship between frequency of any NSAIDs use (year) and gastric cancer risk. Data were modeled with restricted cubic spline models, where - - - - represents the linear trend.

We observed a similar GC risk in studies with more frequency of NSAIDs use compared with studies with less frequency (RR=0.77 for ≥7/week VS RR=0.74 for < 5 years, Table 2 ). There were non-linear relationships between frequency of any NSAIDs use and aspirin use and GC risk (any NSAIDs: P for non-linearity<0.01, Figure 3; aspirin: P for non-linearity<0.01) and a linear relationship in frequency of non-aspirin NSAIDs (P for non-linearity=0.38, P for linear trend<0.01). The chemopreventive effect of any NSAIDs and aspirin use for GC risk may be maximal at 5/week. A monotonically decreasing trend was observed for frequency of less than 5/week, but the inverse relationship was attenuated gradually for frequency of more than 5/week. In addition, we also evaluated the potential relationships in a separate analysis with a linear model and the results showed a decreased trend of GC risk as per 2/week increase in frequency of any NSAIDs (RR=0.90, 95%CI=0.85-0.96) and aspirin (RR=0.90, 95%CI=0.85-0.97).

### NSAIDs use and non-cardia GC

**Figure 4:**
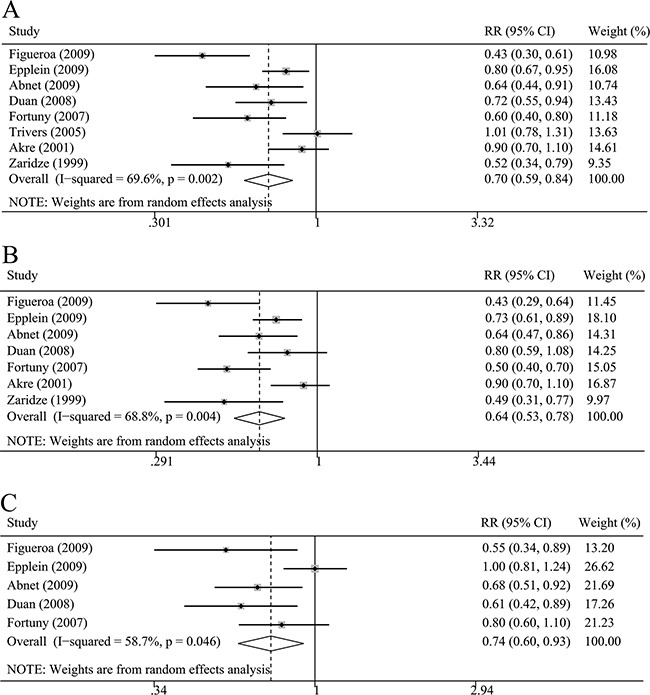
The relative risk (RR) was summarized for the relationship between NSAIDs use and non-cardia gastric cancer risk **A**. Any NSAIDs use and non-cardia gastric cancer. **B**. Aspirin use and non-cardia gastric cancer. (C): Non-aspirin NSAIDs use and non-cardia gastric cancer.

Any NSAIDs use had a chemopreventive effect for non-cardia GC risk (RR=0.70, 96%CI=0.59-0.84, Figure 4). In the term of medication type, aspirin use and non-aspirin NSAIDs use were associated with non-cardia GC (aspirin: RR=0.64, 95%CI=0.53-0.78; non-aspirin NSAIDs: RR= 0.74, 95%CI=0.60-0.93, Figure 4). Subgroup analysis based on the study design, sample size, and country could obtain similar trend, confirming the stability of our results (Table 2).

Longer duration of NSAIDs use had a lower non-cardia GC risk compared with studies with shorter duration (≥ 5 years VS < 5 years, Table 2 ). There were insignificant nonlinear and significant linear relationships between duration of any NSAIDs use and aspirin use and non-cardia GC risk with a decreased trend of non-cardia GC risk as per 2 year increase in duration (any NSAIDs: P for non-linearity=0.07, P for linear trend<0.01, RR=0.83, 95%CI=0.72-0.96; aspirin: P for non-linearity=0.50, P for linear trend<0.01, RR=0.87, 95%CI=0.81-0.93).

### NSAIDs use and cardia GC

Our results indicated that NSAIDs was not associated with cardia GC risk (any NSAIDs: RR= 0.93, 95%CI=0.82-1.05; aspirin: RR= 0.82, 95%CI=0.61-1.11; non-aspirin NSAIDs: RR=0.92, 95%CI=0.78-1.09) (Supplementary Figure S3). Subgroup analysis in any NSAIDs group, aspirin subgroup and non-aspirin NSAIDs subgroup based on study design, sample size and country showed that NSAIDs use did not reduce the risk of cardia GC risk (Table 2). The results of dose-response analysis also showed that increase in duration and frequency of NSAIDs use was not associated with cardia GC risk.

### Assessment of publication bias

The results of Begg's and Egger's tests showed no evidence of publication bias, except in the relationship between non-aspirin NSAIDs and non-cardia GC (P_Begg's_ = 0.09, P_Egger's_ = 0.02) (Supplementary Table S4, Supplementary Figure S4). And the trim-and-fill analysis indicated that publication bias could not substantially impact on the results for the relationship between non-aspirin NSAIDs and non-cardia GC (RR=0.86, 95%CI=0.76-0.97).

## DISCUSSION

Gastric cancer (GC) is a global health problem with unfavorable prognosis [1]. Thus, effective prevention strategies for GC were important for public health. Recently, body of evidence has reported that NSAIDs have protective effects on numerous solid cancer risks [36–38]. However, there are no general agreements about the chemopreventive effect of NSAIDs in GC. Moreover, it remains unclear that whether the chemopreventive effect is different according to differences of tumor site, medication type, and duration and frequency of NSAIDs use.

Our results of both overall and subgroup analysis indicated that any NSAIDs, aspirin, and non-aspirin NSAIDs use could reduce GC risk, especially for non-cardia GC risk. In addition, dose-response analysis indicated decreased trend of GC risk as per 2 years increase in duration of any NSAIDs use and aspirin use, with insignificant nonlinear dose-response relationships. There were nonlinear relationships between frequency of any NSAIDs use and aspirin use and GC risk, and a monotonically decreasing trend was observed only for frequency of less than 5/week, thus the chemopreventive effect of any NSAIDs and aspirin use may be maximal at 5/week.

There were several possible mechanisms responsible for the chemopreventive effect of NSAIDs. The main anti-cancer mechanism was attributed to the blockade of COX-2 pathway because expression of COX-2 was associated with gastric carcinogenesis through promotion of cell proliferation, inhibition of apoptosis, and induction of angiogenesis [6, 39]. Indeed, Sawaoka et al. demonstrated that NSAIDs exerted anti-proliferative activity against GC that overexpressed COX-2 [40]. Nam et al. also reported that NSAIDs could prevent gastric carcinogenesis in mouse models [41]. In addition, some NSAIDs could induce cell death and inhibit proliferation in cells without expression of COX, thus several studies suggested that COX was not the sole target of NSAIDs and other targets may also promote apoptosis [42, 43]. Kopp et al. and Yamamoto et al. demonstrated that NSAIDs could promote apoptosis by inhibiting the activation of nuclear factor κb [44, 45]. Several studies also reported that NSAIDs could prevent carcinogenesis via B-catenin, wnt signalling, tumour necrosis factor, polyamine metabolism and the DNA mismatch repair system[46, 47]. Moreover, NSAIDs could inhibit replication and proliferation of *H. pylori* and potentially increase *H. pylori* clearance [48, 49]. Thus, future studies are needed to elucidate mechanisms of NSAIDs involving in carcinogenesis and investigate whether NSAIDs could be used for the treatment of gastric precancerosis and combination treatment of GC.

Our overall, subgroup, and dose-response analyses consistently showed that NSAIDs use was associated with low risk of non-cardia GC but not of cardia GC. Several clinical studies also found that NSAIDs or aspirin use was associated with only non-cardia GC but not with cardia GC [12, 22, 23, 50]. The potential reason for the different chemopreventive effect of NSAIDs between different anatomic sites was that the risk factors and biological behavior of cardia GC differed greatly from that of non-cardia GC [14–16]. As a definite carcinogen, *H. pylori* infection, which could cause chronic inflammation in stomach and then lead to COX-2 expression and prostaglandin synthesis, was a strong risk factor for non-cardia GC but was not associated with the risk of cardia GC [23, 51]. Indeed, a meta-analysis by Cavaleiro-Pinto et al. reported a no relationship between *H. pylori* and cardia GC with summary RR of 1.08 (95% CI 0.83–1.40)[52]. The incidence of COX-2 overexpression in cardia GC was lower compared with non-cardia GC [53]. Thus, NSAIDs may not markedly reduce the cardia GC risk via inhibiting COX-2. Future studies are needed to investigate the differences of carcinogenesis mechanisms between cardia and non-cardia GC, contributing to primary prevention strategies.

Although NSAIDs use could reduce GC risk, it is still confusing that which type of NSAIDs has a stronger chemopreventive effect for GC. Aspirin was one of the most widely used NSAIDs worldwide. Thus, we conducted subgroup analysis based aspirin and non-aspirin NSAIDs (including many selective NSAIDs that inhibit COX-2 only), and the results indicated that a greater chemopreventive effect of aspirin than that of non-aspirin NSAIDs. Aspirin was non-selective NSAIDs that inhibit COX-1 and COX-2, and experimental study reported that both COX-1 and COX-2 could substantially overexpress in tumor tissues [54]. Therefore, COX-1-dependent effect of NSAIDs might promote chemoprevention for GC besides COX-2-dependent effect. Indeed, Tsuji et al. have demonstrated that COX-1 expressed in tumor could participate in tumor angiogenesis and non-selective NSAIDs had an obviously anti-cancer effect by inhibition of COX-1 [55]. Thus, future studies are needed to explore which NSAIDs type should be used for chemoprevention of GC, considering COX-1-dependent and COX-2-dependent effect simultaneously.

The frequency, duration and dose of NSAIDs use was one of the most important questions, considering efficacy and adverse effects simultaneously. However, the definitions of NSAIDs use in included studies were different. Thus, it was important to evaluate how different definitions of NSAIDs use may impact the results, and we conducted both subgroup analysis and dose-response analysis based on frequency and duration of NSAIDs use. For the frequency of NSAIDs use, subgroup analysis indicated that there was no obviously decreasing trend with increasing frequency of NSAIDs use. Indeed, a nonlinear relationship showed the existence of a threshold effect (5/week) between frequency of NSAIDs use and GC risk, with a monotonically decreasing trend for ≤ 5/week and increasing trend for > 5/week. A similar nonlinear relationship with a threshold effect was also reported in esophageal adenocarcinoma and colorectal cancer [56, 57]. The reasons for the nonlinear relationship were unclear. The chemoprevention of NSAIDs through COX-2-dependent pathway required a much shorter dosing interval because nucleated cells could rapidly resynthesize the COX-2, however, high frequency of NSAIDs use may attenuate their chemopreventive effect due to serious gastric mucosal injury caused by NSAIDs [58, 59]. Moreover, another meaningful and important result was the inversely linear relationship between duration of NSAIDs and GC risk. The risk of non-cardia GC decreased by 17% and 13% for 2 years increment of any NSAIDs and aspirin use, respectively. However, most of included studies did not clearly define the dose of NSAIDs use, and thus the present study could not analyze whether the dose of NSAIDs use would impact the chemopreventive effect. Future studies should assess the risk-benefit profile of NSAIDs use, and explore the optimal duration, frequency and dose of NSAIDs use for chemoprevention for GC.

There were several limitations in this study. First, although the association between duration and frequency of NSAIDs use and GC risk were analyzed, it was still unclear that whether the dose of NSAIDs would impact the chemopreventive effect of NSAIDs. Second, detailed individual information was not available from each published studies. Thus, we could not perfectly adjust all important confounding factors, and confounding factors may underestimate chemopreventive effect of NSAIDs on GC. Third, we could not evaluate the side effects of NSAIDs use, such as gastrointestinal bleeding and ulcer perforation owing to limited data. Thus, whether the chemopreventive benefit of NSAIDs outweighed their side effects was unclear. Moreover, a considerable degree of heterogeneity was observed among the studies and could not be explained completely.

In conclusion, our results indicate that NSAIDs use, including aspirin and non-aspirin NSAIDs, is inversely associated with GC risk, especially for non-cardia GC risk. NSAIDs use may become a feasible approach to prevent non-cardia GC.

## MATERIALS AND METHODS

### Literature search

A literature search was performed for relevant studies using the PubMed and Embase database (up to March 2016). The reference lists of relevant studies and reviews were manually checked for potential studies. The main search terms were as follows: “non-steroidal anti-inflammatory drugs,” “NSAIDs”, “cyclooxygenase-2 inhibitors”, “COX-2 inhibitors”, “aspirin”, “salicylate”, “acetaminophen”, “gastric cancer”, and “stomach cancer”.

### Eligibility criteria

Studies were included if met all the following inclusion criteria: (1) the exposure of interest was any type of NSAIDs, (2) the outcome of interest was GC, including cardia and non-cardia GC, (3) the effect estimates [hazard ratios (HRs), risk ratios (RRs), or odds ratios (ORs)] and corresponding 95% confidence intervals (CIs) could be extracted or calculated from published data, (4) the full text could be obtained. Only the most informative study was included if several duplicated studies were based on the same population. To retain maximum information, some data that only reported in excluded duplicated studies were extracted and added it into the included duplicated study.

### Data extraction and quality assessment

Two reviewers independently extracted following data: first author, publication country and year, study name, study design, definition of NSAIDs use, medication type, tumor site, sample size, duration and frequency of NSAIDs use, effect measures with corresponding 95% CIs, and adjusted variables. The study quality was assessed by Newcastle-Ottawa Scale criteria [60]. Any disagreements on the data extraction were resolved by comprehensive discussion of three authors.

### Statistical analysis

The association between NSAIDs and GC risk was summarized by RRs with 95% CIs. The ORs provided by case-control studies were used as RRs in the pooled process because GC incidence was sufficiently low and the ORs were close to the RRs [61]. If there were various RRs for different population databases in one study, each population database was considered as one independent study. Overall analysis was performed by including all the relevant studies. Subgroup analysis was performed on basis of NSAIDs type, tumor site, duration and frequency of NSAIDs use, study design, quality of study, publication country, and sample size.

A dose–response analysis was used to evaluate the relationship between duration and frequency of NSAIDs use and GC risk (Supplementary File S1) [62, 63]. This analysis required the distribution of cases and non-cases, assigned values of duration and frequency of NSAIDs use, and risk estimates in each category for each study. For intervals, the midpoint of the interval was chosen, and the assigned value of the lowest category was designated as a reference level. For the open-ended upper interval, the value assigned was 20% higher than the low end of the interval [64]. We used restricted cubic splines with three knots at the 25%, 50%, and 75% percentiles of the distribution to examine a potential non-linear dose–response relationship between GC risk and duration and frequency of NSAIDs use [65, 66]. A P-value for non-linearity was calculated by testing the null hypothesis that the regression coefficient of the second spline was equal to zero [65]. A linear dose-response relationship was examined by generalized least-squares regression [63].

The Cochran Q test and the I^2^ statistic were used to measure heterogeneity [67]. If there was substantial heterogeneity, a random effect model was used; otherwise, a fixed effect model was used. Publication bias was evaluated utilizing Egger's and Begg's tests, and trim-and-fill analysis was conducted if there was publication bias [68–70].

All statistical analyses were performed with Stata software (Version 12.0; Stata Corporation, College Station, Texas, USA, 2011). A two-sided P-value <0.05 was considered statistical significance.

## SUPPLEMENTARY FIGURES AND TABLES









## Abbreviations

CI: Confidence Intervals
COX: Cyclooxygenase
GC: Gastric Cancer
H. pylori: Helicobacter pylori
HR: Hazard Ratio
NSAIDs: Non-steroidal Anti-inflammatory Drugs
OR: Odds Ratio
RR: Risk Ratio
