# Supplementary material for: Aspirin and non-steroidal anti-inflammatory drugs use reduce gastric cancer risk: A dose-response meta-analysis
Source: Oncotarget. 2016 Nov 25;8(3):4781–95. doi: 10.18632/oncotarget.13591 (PMC5354871; doi:10.18632/oncotarget.13591)
Supplement: Supplementary file 1 [file oncotarget-08-4781-s001.pdf]

Aspirin and non-steroidal anti-inflammatory drugs use reduce gastric cancer risk: A dose-response meta-analysis

SUPPLEMENTARY FIGURES AND TABLES

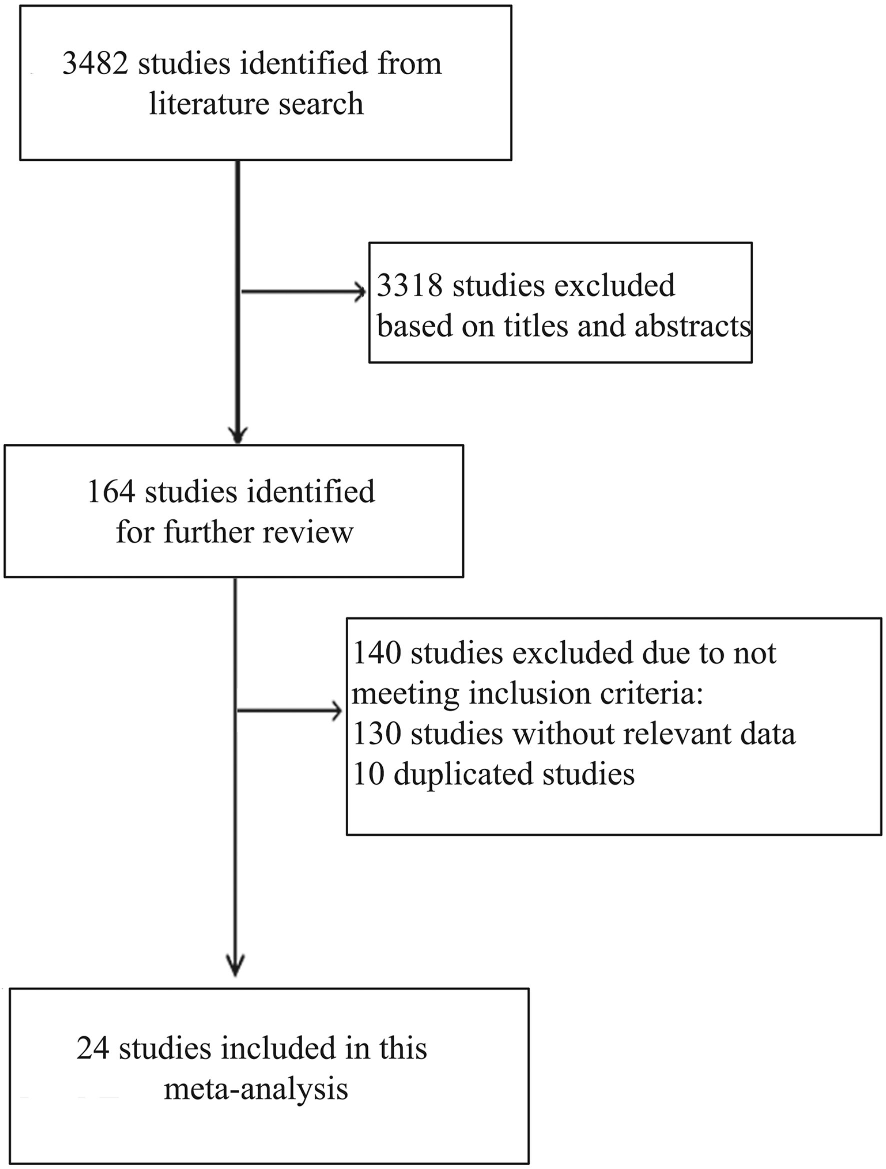

Supplemental Figure S1: Flow diagram showing the study selection process

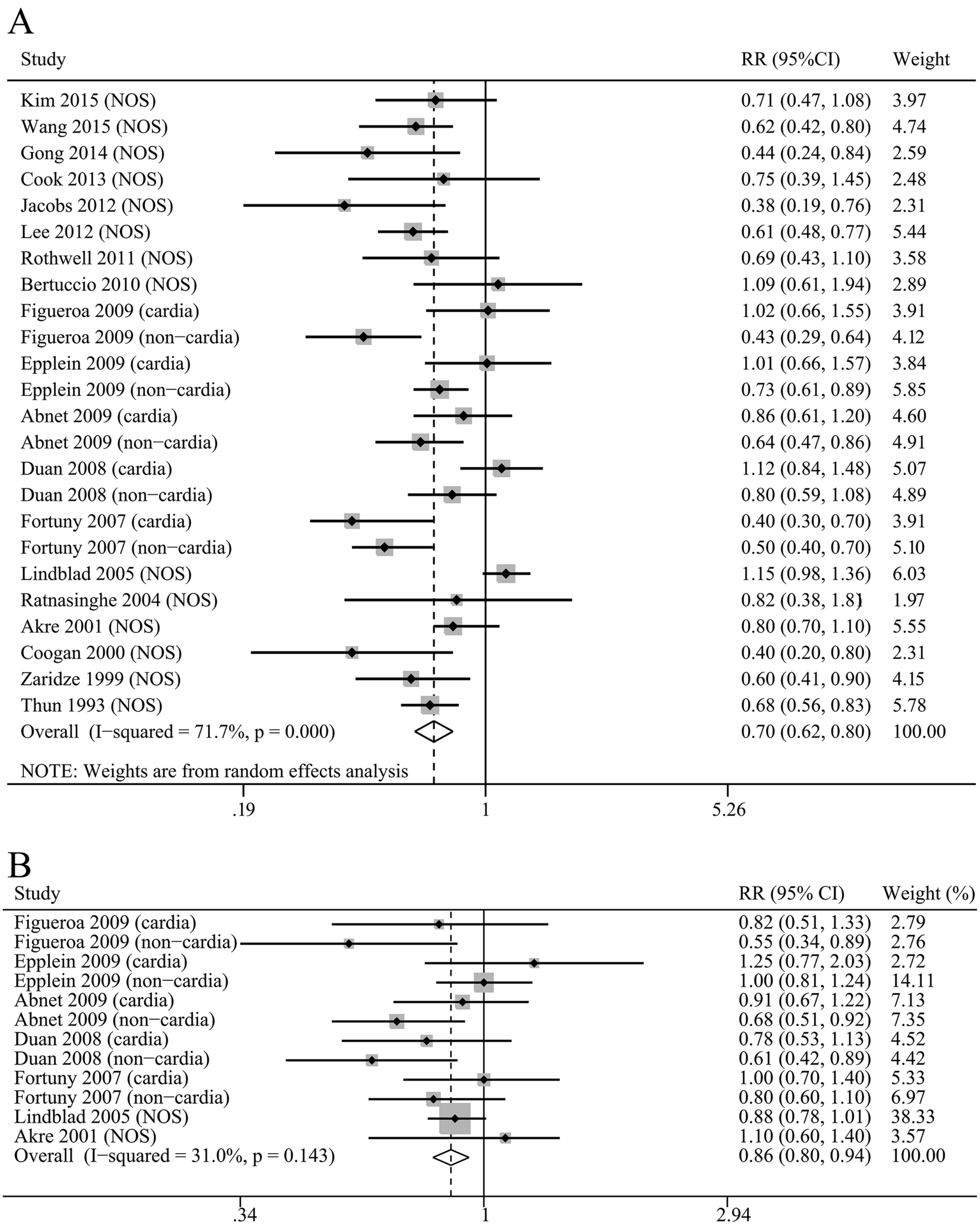

Supplemental Figure S2: The relative risk (RR) was summarized for the relationship between aspirin (A) and non-aspirin NSAIDs use (B) and gastric cancer risk

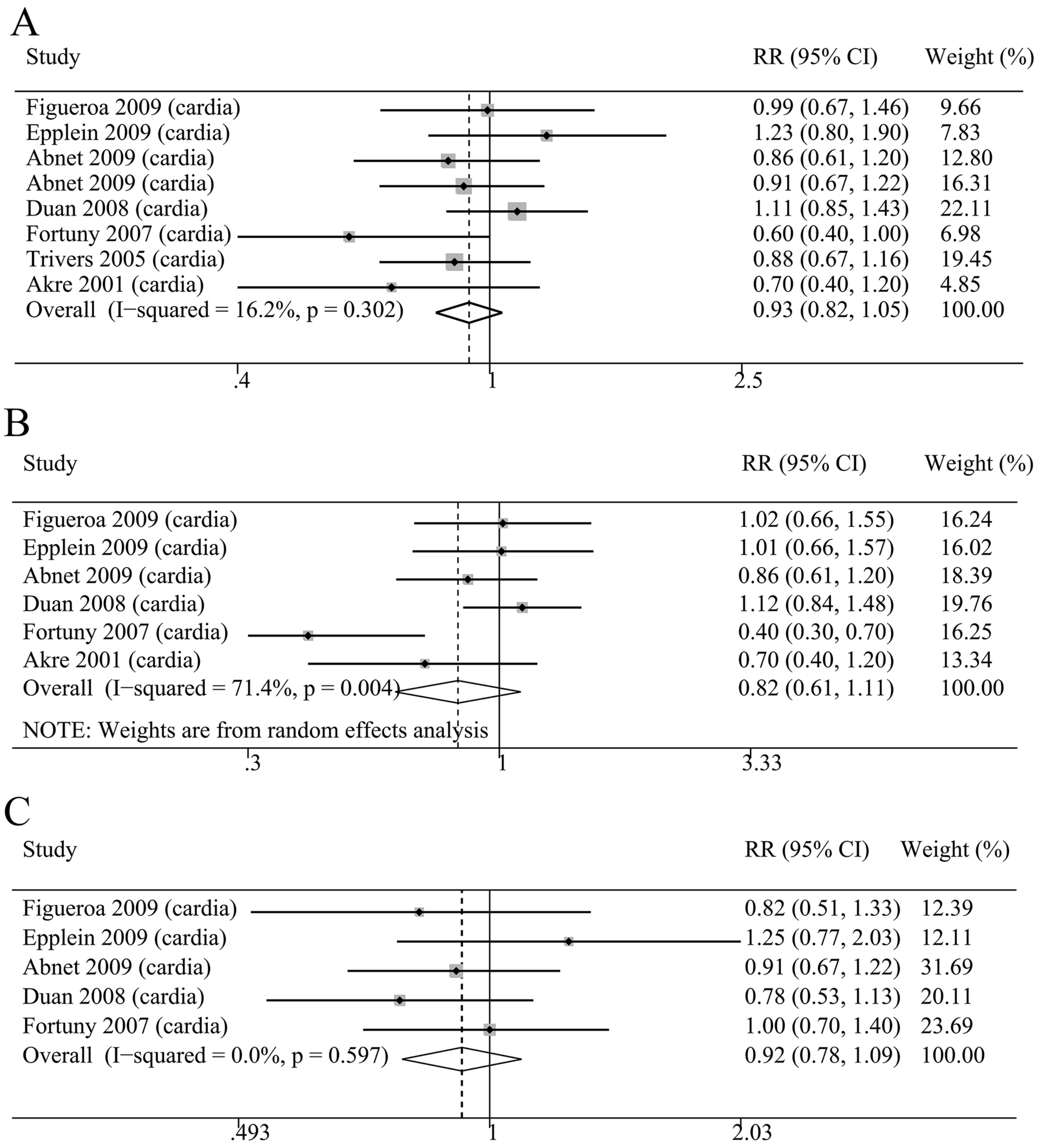

Supplemental Figure S3: The relative risk (RR) was summarized for the relationship between NSAIDs use and cardia gastric cancer risk. A. Any NSAIDs use and cardia gastric cancer. **B.** Aspirin use and cardia gastric cancer. **C.** Non-aspirin NSAIDs use and cardia gastric cancer

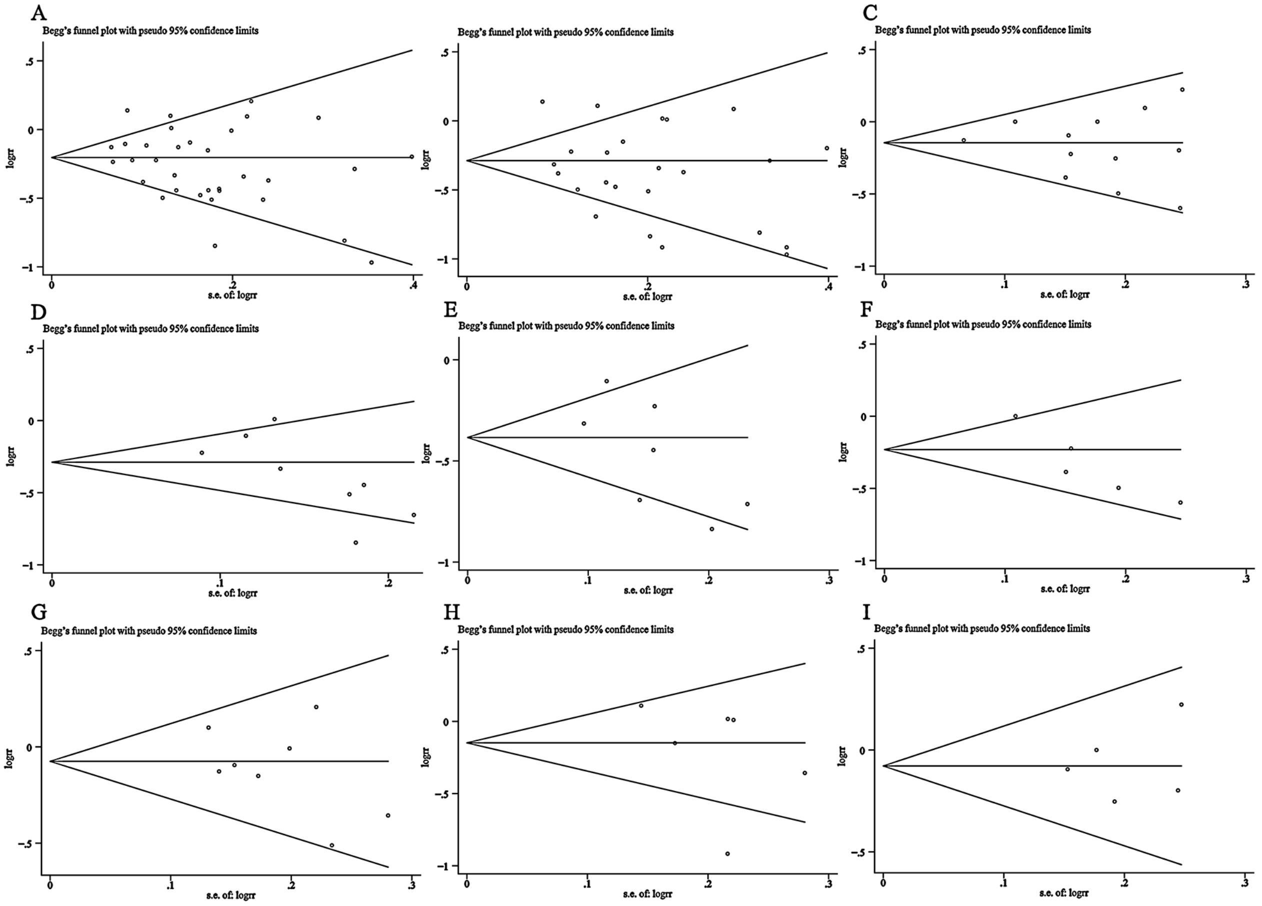

Supplemental Figure S4: Funnel plot for the publication bias. A. Any NSAIDs use and gastric cancer. **B.** Aspirin use and gastric cancer. **C.** Non-aspirin NSAIDs use and gastric cancer. **D.** Any NSAIDs use and non-cardia gastric cancer. **E.** Aspirin use and non-cardia gastric cancer. **F.** Non-aspirin NSAIDs use and non-cardia gastric cancer. **G.** Any NSAIDs use and cardia gastric cancer. **H.** Aspirin use and cardia gastric cancer. **I.** Non-aspirin NSAIDs use and cardia gastric cancer

Supplemental File S1: Estimation procedure for dose-response relationship between duration/frequency of NSAIDs use and gastric cancer

See Supplementary File 1

Supplemental File S2: Variables of duration of NSAIDs use and gastric cancer

See Supplementary File 2

Supplemental File S3: Variables of frequency of NSAIDs use and gastric cancer

See Supplementary File 3

Supplemental Table S4: The results of publication bias

	P (Begg's Test, continuity corrected)	P (Egger's test)
**NSAIDs**		
NOS	0.25	0.06
Non-cardia	0.06	0.05
Cardia	0.27	0.27
**Aspirin**		
NOS	0.44	0.07
Non-cardia	0.55	0.1
Cardia	0.45	0.33
**Non-aspirin**		
NOS	0.84	0.54
Non-cardia	0.09	0.02
Cardia	0.81	0.71

Abbreviations, NOS: Not otherwise specified; NSAIDs: Non-steroidal anti-inflammatory drugs.
