# Supplementary material for: Aspirin and non-steroidal anti-inflammatory drugs use reduce gastric cancer risk: A dose-response meta-analysis
Source: Oncotarget. 2016 Nov 25;8(3):4781–95. doi: 10.18632/oncotarget.13591 (PMC5354871; doi:10.18632/oncotarget.13591)
Supplement: Supplementary file 2 [file oncotarget-08-4781-s002.doc]

**Supplemental File S1: Estimation procedure for dose-response relationship between duration/frequency of NSAIDs use and gastric cancer**

**Step 1:** Data extraction

| **Article** | **Study** | **Type** | **Drug type** | **GC type** | **Year of use** | **Assigned value(dose)** | **Cases** | **N** | **RR** | **Lci** | **Uci** |
| --- | --- | --- | --- | --- | --- | --- | --- | --- | --- | --- | --- |
| Wang | 1 | Case-control | Aspirin | GC NOS | 0 | 0 | 128 | 385 | 1 |  |  |
| Wang | 1 | Case-control | Aspirin | GC NOS | <5 years | 2.5 | 34 | 105 | 0.65 | 0.41 | 1.03 |
| Wang | 1 | Case-control | Aspirin | GC NOS | ≥5 years | 6 | 13 | 35 | 0.67 | 0.43 | 1.21 |
| Bertuccio | 2 | Case-control | Aspirin | GC NOS | 0 | 0 | 208 | 705 | 1 |  |  |
| Bertuccio | 2 | Case-control | Aspirin | GC NOS | <5 years | 2.5 | 12 | 37 | 1.23 | 0.58 | 2.62 |
| Bertuccio | 2 | Case-control | Aspirin | GC NOS | ≥5 years | 6 | 8 | 24 | 1.01 | 0.41 | 2.46 |
| … | … | … | … | … | … | … | … | … | … | … | … |

**Step 2:** Description of data

Article: First author of each study

Study: A numeric indicator variable that assumes the same value across correlated log relative risks within a study

Type: Design type of each study; taking value 1 for case-control data, 2 for incidence-rate data, and 3 for cumulative incidence data

Drug type: Type of NSAIDs use

GC type: Type of gastric cancer according to tumor site

Year/Frequency of use: Duration/ Frequency of NASIDs use

Assigned value (dose): Assigned value of duration of NSAIDs use in each category for each study; designating the lowest category as a reference level

Cases: The number of cases in each category for each study

N: The number of sample size in each category for each study; controls plus cases for case-control data; or the total person-time for incidence-rate data; or the total number of persons for cumulative incidence data

RR: Risk ratios in each category for each study

Lci: Lower limit of 95% confidence intervals of RR

Uci: Upper limit of 95% confidence intervals of RR

**Step 3:** Dose-response analysis using glst commands in Stata software (Version 12.0; Stata Corporation, College Station, Texas, USA, 2011)

1. generating logrr and standard errors of logrr

gen double logrr = log(rr)

gen double loglci = log(lci)

gen double loguci = log(uci)

gen double se= ((loguci- loglci)/(2*invnorm(.975)))

1. Fixed-effect/random-effect dose-response model assuming linearity, and relative ratio for dose* incremental unit

glst logrr dose , se(se) cov(n cases) pfirst(study type) ts(f/r)

lincom dose* , eform

1. Assessing non-linearity for the dose-response relationship using restricted cubic splines with three knots

capture drop doses*

_pctile dose , percentile(25 50 75)

ret list

mkspline doses = dose , knots(`=r(r1)' `=r(r2)' `=r(r3)') cubic displayknots

glst logrr doses* , se(se) cov(n cases) pfirst(study type)

testparm doses2

1. Generating dose–response figure between duration/frequency of NSAIDs use and gastric cancer risk. Relative risk (RR; ———) and the corresponding 95% confidence intervals (CI; — — —) were summarized for the dose–response relationship between duration/frequency of NSAIDs use and gastric cancer risk. Data were modeled with restricted cubic spline models, where - - - - represents the linear trend.

glst logrr dose , se(se) cov(n cases) pfirst(study type) ts(f/r)

predictnl lrr_lin = _b[dose]*dose

gen rr_lin = exp(lrr_lin)

glst logrr doses*, se(se) cov(n cases) pfirst(study type) fixed/random

predictnl logrrwithref = _b[doses1]*doses1 + _b[doses2]*doses2, ci(lo hi)

gen rrwithref = exp(logrrwithref)

gen lbwithref = exp(lo)

gen ubwithref = exp(hi)

twoway (line lbwithref ubwithref rrwithref dose, sort lp(longdash longdash l ) lc(black black black) ) (line rr_lin dose, sort lp(shortdash) lc(black) ) , scheme(s1mono) ylabel(0.5(0.1)1.0, angle(horiz) format(%3.2fc)) xlabel(0(1)12) legend (off) ytitle("Relative Risk", margin(right)) xtitle("duration/frequency of NSAIDs use " , margin(top_bottom) ) yscale(log) plotregion(style(none))
