# Supplementary material for: Aspirin and non-steroidal anti-inflammatory drugs use reduce gastric cancer risk: A dose-response meta-analysis
Source: Oncotarget. 2016 Nov 25;8(3):4781–95. doi: 10.18632/oncotarget.13591 (PMC5354871; doi:10.18632/oncotarget.13591)
Supplement: Supplementary file 3 [file oncotarget-08-4781-s003.docx]

**Supplemental Table S2: Variables of duration of NSAIDs use and gastric cancer**

| **Article** | **Country** | **Study type** | **Drug type** | **GC type** | **Year of use** | **Assigned value** | **RR[95%CI]** |
| --- | --- | --- | --- | --- | --- | --- | --- |
| Wang | China | CCS | Aspirin | GC NOS | 0 year | 0 | 1 |
|  |  |  | Aspirin | GC NOS | <5 year | 2.5 | 0.65[0.41-1.03] |
|  |  |  | Aspirin | GC NOS | ≥5 year | 6 | 0.67[0.43-1.21] |
| Bertuccio | Italy | CCS | Aspirin | GC NOS | 0 year | 0 | 1 |
|  |  |  | Aspirin | GC NOS | <5 year | 2.5 | 1.23[0.58-2.62] |
|  |  |  | Aspirin | GC NOS | ≥5 year | 6 | 1.01[0.41-2.46] |
| Figueroa | USA | CCS | Aspirin | Cardia | 0 year | 0 | 1 |
|  |  |  | Aspirin | Cardia | <5 year | 2.5 | 0.78[0.48-1.28] |
|  |  |  | Aspirin | Cardia | 5-9 year | 7 | 1.18[0.63-2.18] |
|  |  |  | Aspirin | Cardia | ≥10 year | 12 | 0.84[0.48-1.46] |
| Figueroa | USA | CCS | Aspirin | Non-cardia | 0 year | 0 | 1 |
|  |  |  | Aspirin | Non-cardia | <5 year | 2.5 | 0.61[0.39-0.97] |
|  |  |  | Aspirin | Non-cardia | 5-9 year | 7 | 0.56[0.30-1.03] |
|  |  |  | Aspirin | Non-cardia | ≥10 year | 12 | 0.48[0.27-0.82] |
| Figueroa | USA | CCS | Non-aspirin | Cardia | 0 year | 0 | 1 |
|  |  |  | Non-aspirin | Cardia | <5 year | 2.5 | 0.70[0.38-1.30] |
|  |  |  | Non-aspirin | Cardia | ≥5 year | 6 | 0.95[0.45-2.01] |
| Figueroa | USA | CCS | Non-aspirin | Non-cardia | 0 year | 0 | 1 |
|  |  |  | Non-aspirin | Non-cardia | <5 year | 2.5 | 0.59[0.33-1.04] |
|  |  |  | Non-aspirin | Non-cardia | ≥5 year | 6 | 0.48[0.21-1.09] |
| Epplein | USA | Cohort | Non-aspirin | Cardia | 0 year | 0 | 1 |
|  |  |  | Non-aspirin | Cardia | ≤1 year | 0.5 | 0.90[0.37-2.19] |
|  |  |  | Non-aspirin | Cardia | 2-5 year | 3.5 | 1.10[0.49-2.46] |
|  |  |  | Non-aspirin | Cardia | ≥6 year | 7.2 | 1.30[0.62-2.71] |
| Epplein | USA | Cohort | Non-aspirin | Non-cardia | 0 year | 0 | 1 |
|  |  |  | Non-aspirin | Non-cardia | ≤1 year | 0.5 | 1.51[1.11-2.04] |
|  |  |  | Non-aspirin | Non-cardia | 2-5 year | 3.5 | 0.95[0.66-1.36] |
|  |  |  | Non-aspirin | Non-cardia | ≥6 year | 7.2 | 1.00[0.70-1.42] |
| Duan | USA | CCS | Aspirin | Cardia | 0 year | 0 | 1 |
|  |  |  | Aspirin | Cardia | <5 year | 2.5 | 1.30[0.88-1.94] |
|  |  |  | Aspirin | Cardia | ≥5 year | 6 | 0.95[0.63-1.42] |
| Duan | USA | CCS | Aspirin | Non-cardia | 0 year | 0 | 1 |
|  |  |  | Aspirin | Non-cardia | <5 year | 2.5 | 1.00[0.67-1.49] |
|  |  |  | Aspirin | Non-cardia | ≥5 year | 6 | 0.58[0.36-0.92] |
| Duan | USA | CCS | Non-aspirin | Cardia | 0 year | 0 | 1 |
|  |  |  | Non-aspirin | Cardia | <5 year | 2.5 | 0.74[0.47-1.17] |
|  |  |  | Non-aspirin | Cardia | ≥5 year | 6 | 0.86[0.44-1.67] |
| Duan | USA | CCS | Non-aspirin | Non-cardia | 0 year | 0 | 1 |
|  |  |  | Non-aspirin | Non-cardia | <5 year | 2.5 | 0.63[0.41-0.97] |
|  |  |  | Non-aspirin | Non-cardia | ≥5 year | 6 | 0.54[0.24-1.20] |
| Duan | USA | CCS | NSAIDs | Cardia | 0 year | 0 | 1 |
|  |  |  | NSAIDs | Cardia | <5 year | 2.5 | 0.66[0.42-1.06] |
|  |  |  | NSAIDs | Cardia | ≥5 year | 6 | 0.50[0.31-0.82] |
| Duan | USA | CCS | NSAIDs | Non-cardia | 0 year | 0 | 1 |
|  |  |  | NSAIDs | Non-cardia | <5 year | 2.5 | 0.45[0.30-0.68] |
|  |  |  | NSAIDs | Non-cardia | ≥5 year | 6 | 0.34[0.22-0.55] |
| Lindblad | Sweden | CCS | Non-aspirin | GC NOS | 0 year | 0 | 1 |
|  |  |  | Non-aspirin | GC NOS | <3 year | 1.5 | 0.87[0.73-1.05] |
|  |  |  | Non-aspirin | GC NOS | ≥3 year | 3.6 | 0.65[0.44-0.94] |
| Lindblad | Sweden | CCS | Aspirin | GC NOS | 0 year | 0 | 1 |
|  |  |  | Aspirin | GC NOS | <3 year | 1.5 | 1.21[0.98-1.49] |
|  |  |  | Aspirin | GC NOS | ≥3 year | 3.6 | 1.09[0.82-1.45] |
| Nomura | USA | CCS | NSAIDs | GC NOS | 0 year | 0 | 1 |
|  |  |  | NSAIDs | GC NOS | <3 year | 1.5 | 0.60[0.40-1.00] |
|  |  |  | NSAIDs | GC NOS | ≥3 year | 3.6 | 0.70[0.40-1.10] |

Abbreviations, CCS: Case-control study; CI: Confidence interval; GC: Gastric cancer; NOS: Not otherwise specified; NSAIDs: Non-steroidal anti-inflammatory drugs; RR: Risk ratio; USA: the United States of America
