# Supplementary material for: Aspirin and non-steroidal anti-inflammatory drugs use reduce gastric cancer risk: A dose-response meta-analysis
Source: Oncotarget. 2016 Nov 25;8(3):4781–95. doi: 10.18632/oncotarget.13591 (PMC5354871; doi:10.18632/oncotarget.13591)
Supplement: Supplementary file 4 [file oncotarget-08-4781-s004.docx]

**Supplemental Table S3: Variables of frequency of NSAIDs use and gastric cancer**

| **Article** | **Country** | **Study type** | **Drug type** | **GC type** | **Frequency of use** | **Assigned value** | **RR[95%CI]** |
| --- | --- | --- | --- | --- | --- | --- | --- |
| Wang | China | CC | Aspirin | GC NOS | <1/week | 0 | 1 |
|  |  |  | Aspirin | GC NOS | 1-6/week | 3.5 | 0.52[0.32-0.89] |
|  |  |  | Aspirin | GC NOS | ≥7/week | 8.4 | 0.54[0.29-0.84] |
| Figueroa | USA | CC | Aspirin | Cardia | 0 | 0 | 1 |
|  |  |  | Aspirin | Cardia | <7/week | 3.5 | 0.77[0.42-1.43] |
|  |  |  | Aspirin | Cardia | 7/week | 7 | 0.98[0.64-1.50] |
|  |  |  | Aspirin | Cardia | >7/week | 9.6 | 0.54[0.21-1.40] |
| Figueroa | USA | CC | Aspirin | Non-cardia | 0 | 0 | 1 |
|  |  |  | Aspirin | Non-cardia | <7/week | 3.5 | 0.41[0.23-0.75] |
|  |  |  | Aspirin | Non-cardia | 7/week | 7 | 0.52[0.34-0.80] |
|  |  |  | Aspirin | Non-cardia | >7/week | 9.6 | 1.05[0.53-2.10] |
| Figueroa | USA | CC | Non-aspirin | Cardia | 0 | 0 | 1 |
|  |  |  | Non-aspirin | Cardia | <7/week | 3.5 | 0.67[0.30-1.50] |
|  |  |  | Non-aspirin | Cardia | ≥7/week | 8.4 | 0.92[0.51-1.64] |
| Figueroa | USA | CC | Non-aspirin | Non-cardia | 0 | 0 | 1 |
|  |  |  | Non-aspirin | Non-cardia | <7/week | 3.5 | 0.47[0.21-1.04] |
|  |  |  | Non-aspirin | Non-cardia | ≥7/week | 8.4 | 0.57[0.32-1.03] |
| Duan | USA | CC | Aspirin | Cardia | <2/week | 1 | 1 |
|  |  |  | Aspirin | Cardia | 2-7 /week | 4 | 1.10[0.64-1.90] |
|  |  |  | Aspirin | Cardia | ≥7 /week | 8.4 | 1.12[0.8-1.58] |
| Duan | USA | CC | Aspirin | Non-cardia | <2/week | 1 | 1 |
|  |  |  | Aspirin | Non-cardia | 2-7 /week | 4 | 0.97[0.57-1.65] |
|  |  |  | Aspirin | Non-cardia | ≥7 /week | 8.4 | 0.71[0.49-1.04] |
| Duan | USA | CC | Non-aspirin | Cardia | <2/week | 1 | 1 |
|  |  |  | Non-aspirin | Cardia | 2-7 /week | 4 | 0.40[0.12-1.34] |
|  |  |  | Non-aspirin | Cardia | ≥7 /week | 8.4 | 0.79[0.52-1.19] |
| Duan | USA | CC | Non-aspirin | Non-cardia | <2/week | 1 | 1 |
|  |  |  | Non-aspirin | Non-cardia | 2-7 /week | 4 | 1.28[0.58-2.85] |
|  |  |  | Non-aspirin | Non-cardia | ≥7 /week | 8.4 | 0.51[0.33-0.79] |
| Duan | USA | CC | NSAID | Cardia | <2/week | 1 | 1 |
|  |  |  | NSAID | Cardia | 2-7 /week | 4 | 1.05[0.62-1.77] |
|  |  |  | NSAID | Cardia | ≥7 /week | 8.4 | 1.10[0.80-1.48] |
| Duan | USA | CC | NSAID | Non-cardia | <2/week | 1 | 1 |
|  |  |  | NSAID | Non-cardia | 2-7 /week | 4 | 0.95[0.58-1.57] |
|  |  |  | NSAID | Non-cardia | ≥7 /week | 8.4 | 0.63[0.46-0.88] |
| Akre | Sweden | CC | Aspirin | GC NOS | 0/week | 0 | 1 |
|  |  |  | Aspirin | GC NOS | <7 /week | 3.5 | 0.80[0.60-1.00] |
|  |  |  | Aspirin | GC NOS | ≥7 /week | 8.4 | 0.60[0.30-1.10] |
| Thun | USA | Cohort | Aspirin | GC NOS | 0 | 0 | 1 |
|  |  |  | Aspirin | GC NOS | 1-3/week | 2 | 0.60[0.42-0.85] |
|  |  |  | Aspirin | GC NOS | ≥4/week | 4.8 | 0.53[0.34-0.81] |

Abbreviations, CCS: Case-control study; CI: Confidence interval; GC: Gastric cancer; NOS: Not otherwise specified; NSAIDs: Non-steroidal anti-inflammatory drugs; RR: Risk ratio; USA: the United States of America
